# Freestyle perforator flap in earlobe reconstruction

**DOI:** 10.1186/s12893-022-01846-y

**Published:** 2022-11-18

**Authors:** Mohamed Saad Sadaka, Sherif Abou Bakr Hantash

**Affiliations:** Department of Plastic and Reconstructive Surgery, Faculty of Medicine - Tanta University, Tanta, Egypt

**Keywords:** Perforator, Ear-lobe, Reconstruction

## Abstract

**Background:**

In earlobe reconstruction, it is a challenging task to find a simple one-stage technique that is applicable to the variable configurations of defects and that results in a longstanding naturally appearing earlobe.

**Methods:**

In this article, we present a perforator-based flap design for earlobe reconstruction with preoperative perforator detection by pencil doppler to ensure the maximum vascularity of the used flap. Thirty cases of earlobe reconstruction were performed in the Department of Plastic and Reconstructive Surgery at Tanta University in the period from July 2015 to July 2019 using the technique described in this article.

**Results:**

None of our cases developed necrosis of the flaps. The mean result for the aesthetic evaluation of our cases was 4.7 and the mean patient satisfaction was 4.8 where the maximum score was 5.

**Conclusion:**

freestyle perforator flap with preoperative doppler perforator detection is a safe and reliable method of earlobe reconstruction.

**Supplementary Information:**

The online version contains supplementary material available at 10.1186/s12893-022-01846-y.

## Introduction

The earlobe plays a crucial role in the aesthetic facial appearance and also a functional role through sound gathering [1].

The earlobe is made of fibrofatty tissue covered by skin and lacks cartilage. Earlobe loss may result from acquired or congenital causes. Avulsion wounds, wearing jewelry, murderous assaults, auto accidents, and thermal and chemical burns are some examples of acquired causes. The patient has severe psychological suffering due to earlobe loss [2].

In earlobe reconstruction, it is a challenging task to find a simple one-stage technique that is applicable to the variable configurations of defects and that results in a longstanding naturally appearing earlobe Numerous publications have reported various flap designs, but none of them have described using preoperative doppler perforator detection [3].

Most flaps used for earlobe reconstruction are derived from the adjacent tissues. The pattern of the defect, the vascularity of the surrounding tissues, and the sites of previous scars all influence the choice of the flap. Preauricular, retroauricular, infraauricular, retromandibular, or auricular skin are common sources for flaps in earlobe reconstruction [4].

In this article, we present a perforator-based flap design for earlobe reconstruction with preoperative perforator detection by pencil doppler to ensure the maximum vascularity of the used flap.

## Patients and methods

The authors declare that all methods were carried out in accordance with the relevant guidelines and regulations.

The authors declare that ethical approval was obtained for this study from the institutional ethical committee of Tanta University.

Authors declare that informed consent was obtained from all subjects and/or their legal guardians.

### Study design

A case series study.

### Study population and sampling

The study was carried out from July 2015 to July 2019 at Tanta University's Plastic Surgery Department, Egypt. All patients (n = 30) attended the plastic surgery department were included in the study.

### Inclusion criteria

All patients with earlobe defects of any size and etiology including traumatic defects, post-tumor resection defects, defects due to burns, and congenital defects.

### Exclusion criteria

Any patient with a contraindication to general anesthesia, patients with systemic diseases impairing the wound healing, smokers until they stop smoking for at least 1 month before surgery, and the presence of active inflammation or residual malignancy at the site of planned surgery.

A pencil doppler was used to map the perforators at the location of the intended flap pedicle around the earlobe remnant. Search for the reliable perforator was performed along the anticipated course of the posterior auricular and the superficial temporal arteries. The perforator with the strongest signal was marked and its location was confirmed again intraoperatively after flap elevation by pencil doppler (Additional file 1: Video S1). The flap was designed based on the detected perforator. A paper template of the contralateral normal earlobe was used to help achieve symmetry between the two sides.

**Fig. 1:**
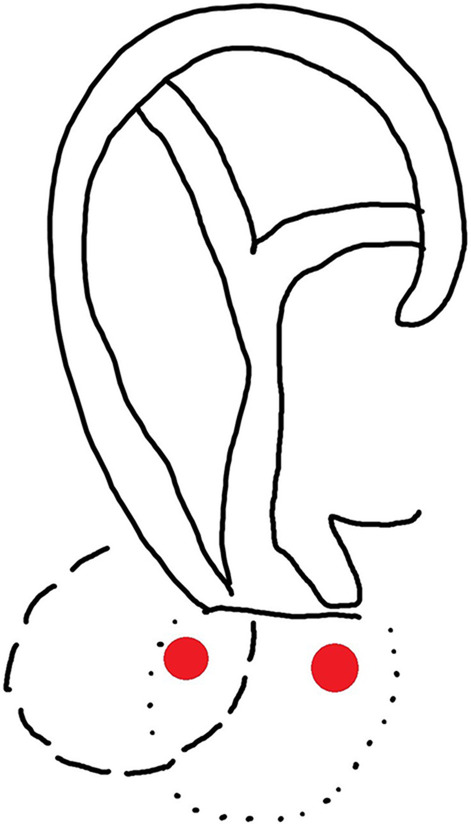
An illustration showing two possible designs of our flap based on the doppler detected perforator (right-sided perforator supplies the flap with dotted outline and left-sided perforator supplies the flap with interrupted line outline)

**Fig. 2:**
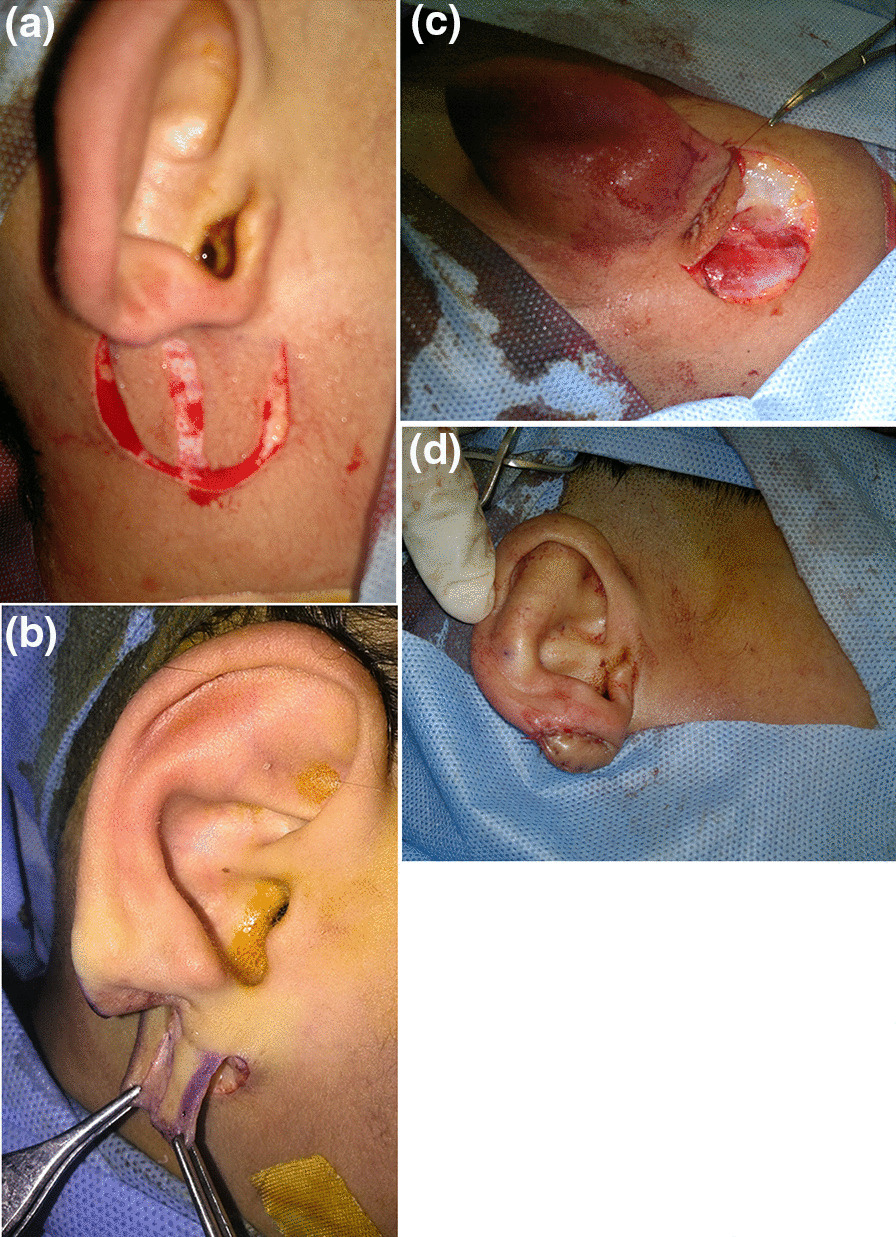
**a** Intraoperative view of deepithelialization at the middle and the flap incision. **b** The flap was elevated. **c** The margins of the de-epithelialized area were sutured to the edges of the earlobe remnant. **d** The outer free border of the flap was then sutured to itself. The secondary defect was closed primarily

All cases were performed under general anesthesia. At the middle of the flap, a 4–6 mm strip of skin was de-epithelialized extending the whole length of the flap. Then the flap was elevated in the subcutaneous plane and the margins of the de-epithelialized area are sutured to the edges of the earlobe remnant (after the edges have been freshened with a No 11 blade). The outer free border of the flap was then sutured to itself with nylon 5/0 suture. The secondary defect was closed primarily with minimal undermining in the subcutaneous plane without the need for a skin graft. No cartilage grafts were used (Figs. 1 and 2).

A self- structured questionnaire sheet was designed for data collection. It was developed grounded on the associated literatures and statistical experts in our college assessed its validity and reliability. It consisted of two sections as follows:

*Section one*: every patient was asked about his satisfaction with following nine items and asked to give each item a score from 1 to 5 where the lower score means poorer satisfaction:

- Satisfaction with shape of auricle.
- Satisfaction with size of auricle.
- Satisfaction with similarity to the normal auricle.
- Does the patient feel that the reconstructed auricle is natural.
- Does the patient receive negative comments from surrounding people regarding his auricle.
- Will the patient undergo the operation again if there is choice.
- Did the surgeon explain the outcome enough for the patient.
- Did the patient receive enough pain relief after surgery.
- Overall satisfaction with the operation.

The level of patient satisfaction with each item was rated on a 5-point Likert scale ranging from poor satisfaction [1] to excellent satisfaction [5]. Scoring of all questions was summed and the mean of total score calculated. Higher scores reflect more patient satisfaction.

Section two: This section is consisted of four items and represent aesthetic evaluation of the reconstructed auricle by two plastic surgeons who are independent from the study:

- Size of the new auricle.
- Shape of the new auricle.
- Scar quality.
- Overall aesthetic evaluation.

The plastic surgeon’ evaluation also was rated on a 5-point Likert scale ranging from poor outcome [1] to excellent outcome [5]. Scoring of all questions was summed and the mean of total score calculated. The higher score means more perfect outcome.

Aesthetic plastic surgeon’ evaluation and assessment of patient satisfaction were performed 6 months postoperatively to allow a proper period of scar maturation.

### Statistical analysis

Statistical Package for Social Sciences (SPSS IBM Chicago, version 23) was used to analyze data of the study. Descriptive presentations were done for all variables of the study and total score measured with mean and standard deviation.

## Results (Figs. 3, 4, 5)

**Fig. 3:**
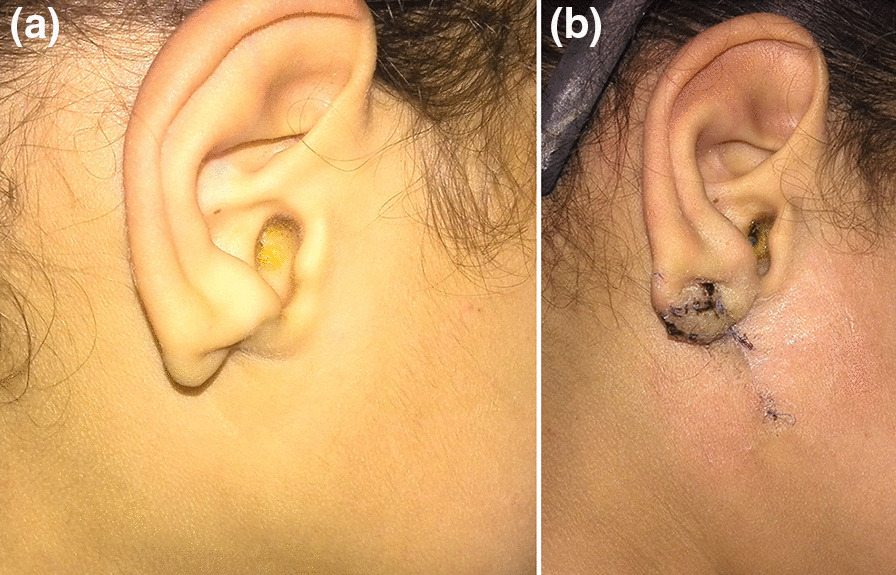
**a** Seven years old girl preoperative lateral view of the earlobe defect. **b** Postoperative lateral view

**Fig. 4:**
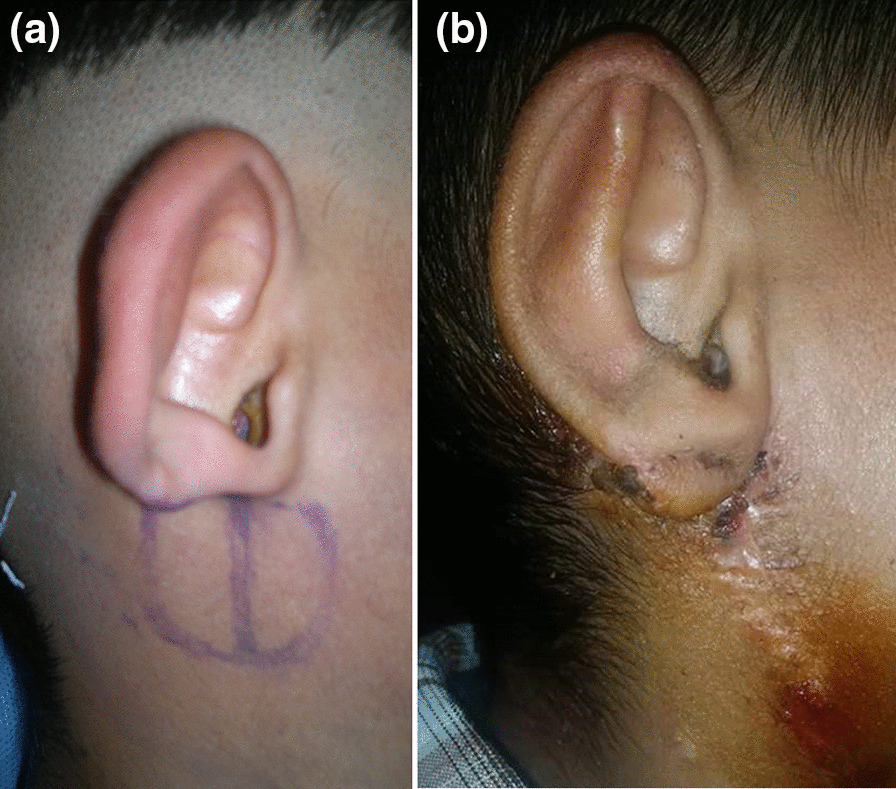
**a** Fifteen years old male preoperative view of the defect and the flap marking. **b** Postoperative lateral view

**Fig. 5:**
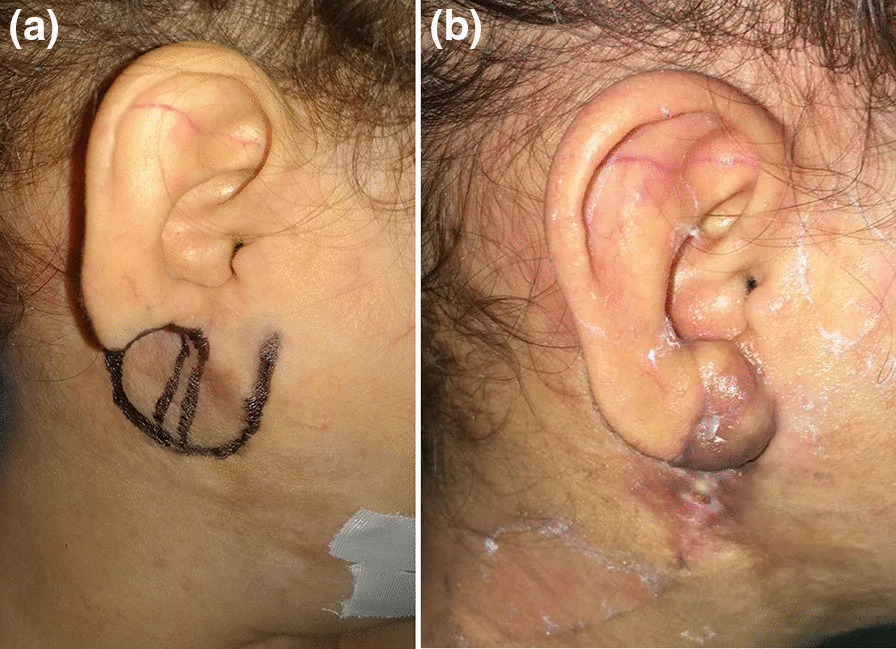
**a** Six years old girl preoperative lateral view of the defect and the flap marking. **b** Postoperative lateral view

The procedure described in this article was used in thirty cases of earlobe reconstruction at Tanta University's Department of Plastic and Reconstructive Surgery between July 2015 and July 2019. No partial or total necrosis developed in any of our flaps.

Eighteen of our cases (60%) were males and twelve cases (40%) were females. The age of our patients ranged from 5 to 47 years (average 22.7 years). Regarding the etiology of earlobe defects in our cases; 23 cases were caused by trauma, 6 cases were due to surgical resection of earlobe lesions and one case was due to congenital absence of earlobe.

No wound complications were encountered in our cases except for two cases of minor wound disruption of the donor site wound and both cases were successfully managed conservatively with resulting complete wound healing.

All patients were scheduled for postoperative follow up visits after 1 week, 2 weeks, 1 month, 3 months, 6 months, 1 year after surgery and then yearly. All cases completed at least 30 months of follow up after surgery.

The mean patient satisfaction in our cases was 4.8 (Table 1). The mean result for the plastic surgeon’ aesthetic evaluation of our cases was 4.7 (Table 2). The aesthetic result of the reconstructed auricle that was achieved immediately postoperative was maintained in all cases along the whole postoperative follow-up course.

**Table 1:** The results of patient satisfaction of our cases

Item of patient satisfaction	Number and percent of patients reported for each score 1–5				
	Score 1	Score 2	Score 3	Score 4	Score 5
Satisfaction with shape of auricle	1 (3.3%)	0 (0%)	1 (3.3)	5 (16.7%)	23 (76.7%)
Satisfaction with size of auricle	0 (0%)	0 (0%)	0 (0%)	5 (16.7%)	25 (83.3%)
Satisfaction with similarity to the normal auricle	1 (3.3%)	0 (0%)	1 (3.3%)	1 (3.3%)	27 (90%)
Do patient feel that the reconstructed auricle is natural	0 (0%)	0 (0%)	1 (3.3%)	5 (16.7%)	24 (80%)
Did patient receive negative comments from surrounding people regarding his auricle	0 (0%)	0 (0%)	1 (3.3%)	3 (10%)	26 (86.7%)
Will the patient undergo the operation again if there is choice	1 (3.3%)	0 (0%)	1 (3.3%)	4 (13.3%)	24 (80%)
Did the surgeon explain the outcome enough for the patient	0 (0%)	0 (0%)	1 (3.3%)	5 (16.7%)	24 (80%)
Did patient receive enough pain relief after surgery	0 (0%)	0 (0%)	0 (0%)	1 (3.3%)	29 (96.7%)
Overall satisfaction with the operation	0 (0%)	1 (3.3%)	1 (3.3%)	5 (16.7%)	23 (76.7%)
Mean score of patient satisfaction	4.8 ± 0.4

**Table 2:** The results of the plastic surgeons’ aesthetic evaluation of our patients

	Surgeon 1 evaluation (number of cases for each score 1–5)					Surgeon 2 evaluation (number of cases for each score 1–5)				
	1	2	3	4	5	1	2	3	4	5
Size of new earlobe	0 (0%)	0 (0%)	4 (13.3%)	6 (20%)	20 (66.7%)	0 (0%)	0 (0%)	0 (0%)	6 (20%)	24 (80%)
Shape of new earlobe	0 (0%)	0 (0%)	2 (6.7%)	3 (10%)	25 (83.3%)	0 (0%)	0 (0%)	1 (3.3%)	3 (10%)	26 (86.7%)
Scar quality	0 (0%)	0 (0%)	6 (20%)	9 (30%)	15 (50%)	0 (0%)	0 (0%)	5 (16.7%)	7 (23.3%)	18 (60%)
Overall appearance of new earlobe	0 (0%)	0 (0%)	2 (6.7%)	5 (16.7%)	23 (76.7%)	0 (0%)	0 (0%)	2 (6.7%)	5 (16.7%)	23 (76.7%)
Mean surgeon aesthetic evaluation score	4.7 ± 0.4									

## Discussion

There are numerous flap designs reported for earlobe repair. A transposition flap from infrauricular skin was described with soft tissue at the flap tip used to add bulk to the repaired earlobe [5]. For larger defects, a combination of an extended postauricular flap with a preauricular flap was reported [6]. The same idea was reported by another author describing the combination of two opposing flaps but from the preauricular and retromandibular regions [7]. Shen et al. described the reconstruction of earlobe defects ranging in size from 5 to 8 mm using two rotation flaps; one from the lower auricle and the other from the infraauricular area with V–Y advancement flap closure of the donor defect [1]. Gavello in 1907, described a bilobed flap; one lobe in the mastoid region and the other in the retroauricular region and his design was modified later by many authors [3, 8, 9]. Other techniques described include postauricular turnover flap with ipsilateral conchal cartilage graft [10], retro auricular anteriorly based flap [11], Infra-auricular bilobed superiorly based Flap [12], and preauricular superiorly based flap [13].

In an anatomical study, all auricles received arterial blood flow from branches of the superficial temporal (STA) and posterior auricular arteries. (PAA). In most auricles (81%), the STA had three branches: superior, middle, and inferior which run perpendicularly to supply the lateral auricular surface [14].

Henoux et al., in another anatomical study of 11 auricles, found that the arterial supply of the auricle is more dependent on the PAA than the STA. They also found that the PAA was observed to ascend in the groove between the cranial aspect of the auricle and the mastoid process in every sample. It terminated on the posterior auricular surface in 5/11 auricles, whereas it continued its course upward to the temporoparietal scalp in 6/11 auricles. In addition to posterior branches for the scalp and mastoid, the PAA gave off 2 to 4 auricular branches with a bottom-up, or horizontal path, toward the margin of the helix [15]. The “middle branch” described by Park was found in 10 dissections (91%) [16]. Besides this branch, the “upper, middle, and lower divisions” scheme was observed inconsistently (63%). Tilotta et al. reported the absence of PAA in two of ten dissected auricles and that in this case, the medial region was supplied by branches from occipital artery [17].

The blood supply to cutaneous tissues arises from the so-called source artery; courses to the skin either directly or indirectly; and includes direct cutaneous vessels, septocutaneous vessels, musculocutaneous perforators, and vessels found above the fascia linked by “choke vessels [18]. The perforator flap has revolutionized the approach to reconstructive flap surgery. Tissues through which the vascular supply for a skin island traverses can be spared to reduce donor-site morbidity and flap bulk [19].

The advantage of the perforator flap design is that it allows the surgeon to divide the skin at the flap’ pedicle, so removing its tethering effect on the flap mobility. The flap remains attached to the donor area only by the supplying perforator. This gives the surgeon more ability to three-dimensionally shape the flap in order to perfectly and smoothly fit into the defect. Additionally, the perforator design allows preservation of the main source artery as the flap is based solely on the skin branch.

The concept of freestyle perforator-flap surgery gives more flexibility in selecting the donor site since flap selection is dependent on the quality and volume of soft tissue required at the recipient site. The preoperative Doppler examination is crucial in free-style flap surgery because it offers essential details about the quality and topography of the perforator vessels [20].

In this study, we use preoperative pencil doppler for the detection of perforators to ensure the inclusion of a reliable perforator at the base of the intended flap design and also intraoperative doppler confirmation of the perforator location at the flap’ pedicle after its elevation. This ensures the maximum vascularity of the flap and aims to decrease the rate of postoperative ischemic flap complications and also allows the application of this flap to large defects and even total earlobe loss as in the case in Fig. 3.

The method of transfer of the flap to the defect was either advancement, rotation, or a combination of both methods. The flap should be designed, as possible, in an area with adequate laxity that allows for primary closure of the donor site. this can be confirmed preoperatively by the pinch test [21].

A similar flap design was described by Gupta and Devendra [22] but without preoperative or intraoperative perforator detection which makes the flap a random one and with a possible risk of postoperative flap necrosis, especially if used on a large number of patients.

A doubled-over Limberg flap was described for earlobe reconstruction in six cases. The authors reported the need for defatting after 4 months and necrosis at the tip of the flap in three (50%) cases [23]. The use of a free-style perforator flap with preoperative doppler detection of the perforator will make the surgeon confident that the blood supply is included within the flap and hence decreasing the risk of flap necrosis and at the same time allowing the surgeon to elevate a flap that is only thick enough to achieve the desired cosmetic effect.

Some authors prefer to perform earlobe reconstruction in two stages to avoid vascular compromise of the flap [24]. Our technique allows one-stage reconstruction without fear of ischemic flap complications. All the previously mentioned studies were conducted on a small number of patients which is not enough to conclude the vascular safety of the described procedure.

## Conclusion

The freestyle perforator flap with preoperative doppler identification of perforators is a safe and reliable technique and provides the surgeon and patient with the additional safety of the inclusion of a reliable vessel at the flap base that allows its use in large defects or even total earlobe loss. It has many other advantages; an excellent aesthetic result with the restoration of earlobe shape and volume and excellent color match and excellent patient satisfaction. In addition, it is a single stage and does not require a skin graft.

## Supplementary Information

**Additional file 1: Video S1**: intraoperative pencil doppler confirmation of the location of the perforator at the flap’ Pedicle.

## Data Availability

All data generated and analyzed during this study are included in this published article (and its Additional files).
